# Beyond the 3′UTR binding–microRNA-induced protein truncation via DNA binding

**DOI:** 10.18632/oncotarget.26023

**Published:** 2018-08-28

**Authors:** Melanie von Brandenstein, Stephan H. Bernhart, Andreas Pansky, Claudia Richter, Tobias Kohl, Martina Deckert, Axel Heidenreich, Peter F. Stadler, Manuel Montesinos-Rongen, Jochen W.U. Fries

**Affiliations:** ^1^ Department of Urology, University Hospital of Cologne, Cologne, Germany; ^2^ Institute of Pathology, University Hospital of Cologne, Cologne, Germany; ^3^ Department of Natural Sciences, University of Applied Sciences Bonn-Rhein-Sieg, Rheinbach, Germany; ^4^ Institute of Neuropathology, University Hospital of Cologne, Cologne, Germany; ^5^ Bioinformatics Group, Department of Computer Science and Interdisciplinary Center of Bioinformatics, Leipzig University, Leipzig, Germany

**Keywords:** Vim3, Mxi-2, miR-498, miR-15, DNA interaction

## Abstract

Here, we present a miR mechanism which is active in the nucleus and is essential for the production of intron included, C-terminal truncated and biologically active proteins, like e.g. Vim3. We exemplified this mechanism by miRs, miR-15a and miR-498, which are overexpressed in clear cell renal carcinoma or oncocytoma.

Both miRs directly interact with DNA in an intronic region, leading to transcriptional stop, and therefore repress the full length version of the pre-mRNA, resulting in intron included truncated proteins (Mxi-2 and Vim3). A computational survey shows that this miR:DNA interactions mechanism may be generally involved in regulating the human transcriptome, with putative interaction sites in intronic regions for over 1000 genes.

In this work, an entirely new mechanism is revealed how miRs can repress full length protein translation, resulting in C-terminal truncated proteins.

## INTRODUCTION

In these times of personalized medicine, the detection of new biomarkers for the identification, differentiation or early diagnosis of diseases is of great importance.

Recently we identified a structural protein, called Vimentin variant 3 (Vim3), which is the only known histological biomarker usable for the differentiation of oncocytoma (benign kidney cancer) and eosinophilic variant of the clear cell renal carcinoma (eRCC). The clear differentiation between oncocytoma and malignant kidney tumor types will be essential in the future for patients’ therapy as well as operative planning, follow-up therapy, and patients’ survival [1].

The identification of Vim3 as a biomarker was initiated by discrepant results of different commercially available antibodies for Vimentin: One directed against epitopes in the C-terminus of the full length Vimentin (V9), the other against epitopes in the rod domain (exon 4–5) of the full length version (3B4). For the V9 antibody, most or nearly all oncocytoma were negative and all RCCs were positive. In contrast, using the 3B4 antibody both tumor types were positive. We found that a published, truncated splice variant of Vimentin (accession number ACA06103.1, called Vim3) was able to explain why these two different antibodies give such discrepant results. Surprisingly, in 2011 Thakkar described the presence of this truncated variant in gliomas [2]. In terms of sequence difference, the C-terminus of Vim3 comes from an intronic part of Vimentin, namely intron 7.

By comparing the amino acid sequences of Vimentin and Vim3 it is noticeable that both are homolog until exon 7, where the Vim3 variant is truncated and ends up with an additional C-terminal ending of 8 amino acids. Interestingly, a biomarker for RCC, Mxi-2, is a truncated version of MAPK14 that follows the same mechanism (patent pending).

Besides proteins, micro RNAs (miRs) are a promising candidate for biomarkers. As an example, we have identified miR-15a to be overexpressed in RCCs tissue [3] and has recently also be shown by Mytsyk *et al.* [4].

In general miRs are short (21-23 nucleotides), non-coding RNAs that play important roles in cell function and development by targeting mRNA sequences of protein-coding transcripts [5–7]. This usually occurs at the 3′untranslated region (UTR) by annealing with the so called miR seed sequence (6–8 nucleotides) and results in either mRNA cleavage or repression of productive translation [5–7]. To date, computational and cloning approaches have discovered several hundred miRs in the human genome. Human miRs are frequently located in fragile sites and genomic regions involved in cancer [8].

In the cytoplasm the Ago2 complex is involved in the interaction of miRs with the target RNA sequences [9, 10]. Furthermore, Ago2 interacts in the cytoplasm with importin 8 which is necessary for the translocation of Ago2 and certain miRs, e.g. miR-21 into the nucleus [11]. In 2014 Gagnon *et al.* demonstrate the localization of miRs in the nucleus and their association with Ago2 [12]. They demonstrate that around 75% of all analyzed miRs shuttle from the cytoplasm into the cell nucleus [12]. So far, he role of nuclear located miRs has been largely ignored and is not well understood [13].

It has been assumed that the nucleus is a possible storage portal for miRs which release from the nucleus in case of e.g. cell stress [13]. Tang *et al.* published in 2012 that in mouse the translocation of miRs into the nucleus is responsible for the post-transcriptional regulation of pri-miRs [14]. Furthermore, it was shown that some miRs can interact with the promoter regions of specific genes and thereby induce transcriptional gene activation [15]. A similar mechanism has been described in a human non-small cell lung cancer cell line [16].

Investigating our tumor collective for mutual miRs for the differentiation of benign and malignant kidney cancer as biomarkers, we identify miR-498 which is overexpressed in oncocytoma and miR-15a which is known to be overexpressed in RCCs tissue as well as in the urine.

We hypothesized that there is a possible relationship between miR translocation into the nucleus and an increased expression of C-terminal truncated, intron included biologically active proteins. Therefore, we compared the C-terminal sequences of both truncated proteins with both miRs of possible interaction sites.

In this work, we identify miR-498 as a biomarker for oncocytoma. We show that it, as well as the biomarker miR-15a, interacts with DNA in the nucleus leading to an isoform switch of Vimentin and MAPK14. Interestingly, this partially intron retention does not lead to nonsense mediated decay, but the resulting C-terminally truncated proteins, Mxi-2 and VIM-3, are biologically active. Furthermore, computational analysis shows that there are many more putative intronic targets of known micro RNAs. Thus, this is probably a common mode of regulation through miRs.

This newly identified mechanism of miR function opens a new field for the biological research area, as the function of most of these identified C-terminally truncated proteins is unknown and increased therefore the portfolio of our known proteins.

## RESULTS

### Markers differentiating kidney tumors

**Figure 1:**
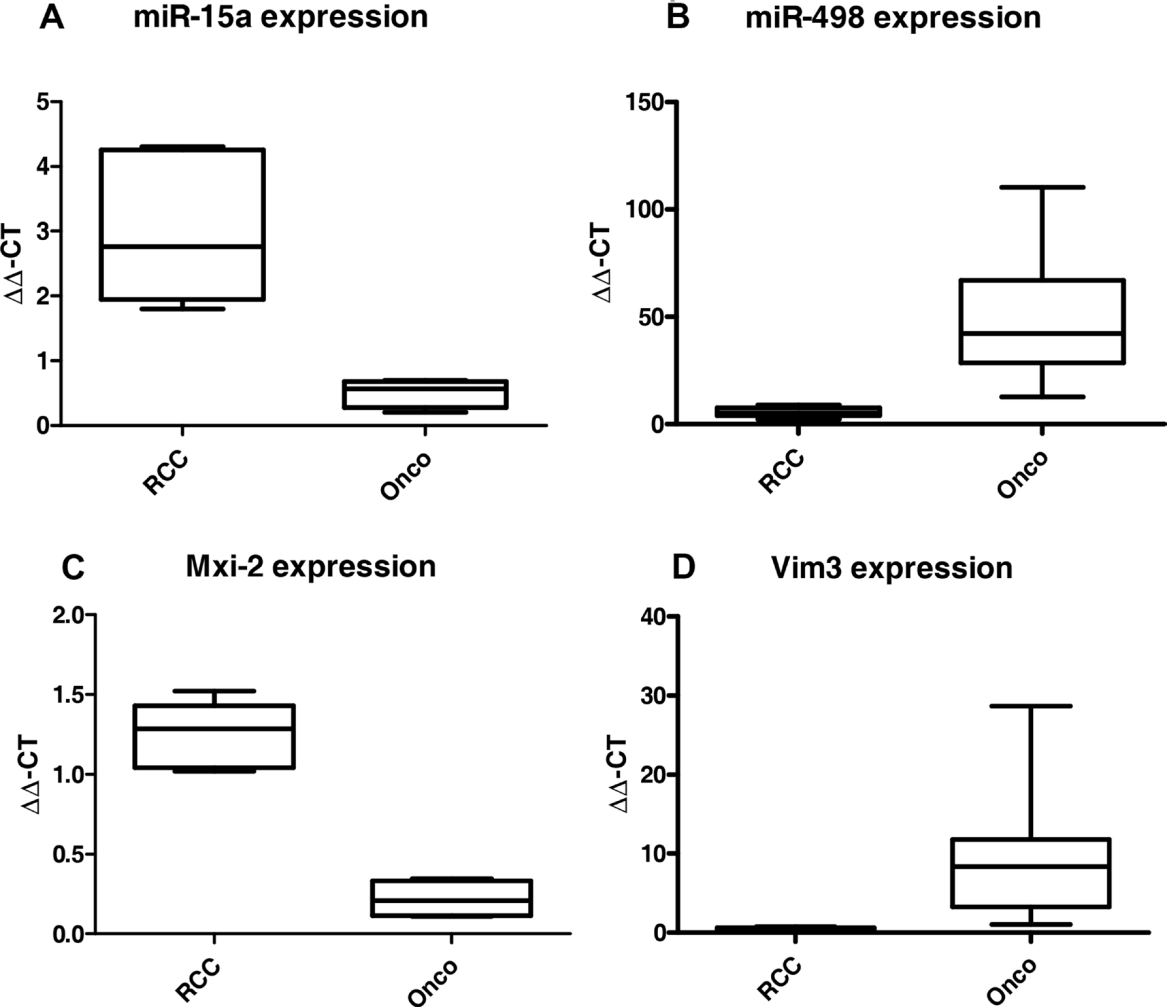
miR levels and expression of the truncated variants in malignant renal cancer (RCC) and benign renal cancer (Onco) qRT-PCR results (ΔΔC_T_ method) of (**A**) miR-15 and (**B**) miR-498 expression in RCC vs Onco, paraffinzed tissue samples. All results were normalized to tumor free kidney tissue. Both miRs show statistically significant (ANOVA) differences between expression in the benign and malignant kidney tumors. (**C**) and (**D**) Expression levels in benign and malignant kidney tumors of the truncated variants of MAPK14 (Mxi-2, C) and Vimentin (Vim3, D). (^***^p < 0.001, ^**^p < 0.01, ^**^p < 0.05).

In 2012 we showed that miR-15a is a biomarker for the differentiation between oncocytoma and malignant variants of kidney cancer in patient’s urine [17]. Re-analyzing our tumor collective, we identified another miR as a possible biomarker overexpressed in oncocytoma, namely the miR-498 (Figure 1). Searching of non-invasive markers essential for the differentiation between benign and malignant kidney tumors, two miRs were identified in the patient’s urine of the two groups. A significant upregulation of both miRs in tissue samples, either in the malignant kidney cancer RCC (Figure 1A) or in the benign Oncocytoma (Figure 1B) could be detected. Oncocytoma overexpress a truncated variant of Vimentin called Vim3 (Figure 1D) [1]. In order to identify additional possible marker proteins, we firstly analysed the single partners of the NF-kB signalling pathway, which is activated in malignant kidney tumors [18]. We recognize a truncated variant of MAPK14, called Mxi-2, being overexpressed in the malignant tumors (Figure 1C).

### miR presence in the nucleus

Based on these data, we hypothesized that the two miRs (mir-15a and miR-498) were responsible for the “truncation” reaction on the DNA level. While the presence of miRs in the nucleus, where they may be involved in different mechanisms like e.g. gene expression regulation has already been described earlier [13, 14, 19], miRNAs are still believed to be mostly active in the cytoplasma. Therefore, we first investigated the cellular location of these miRs.

**Figure 2:**
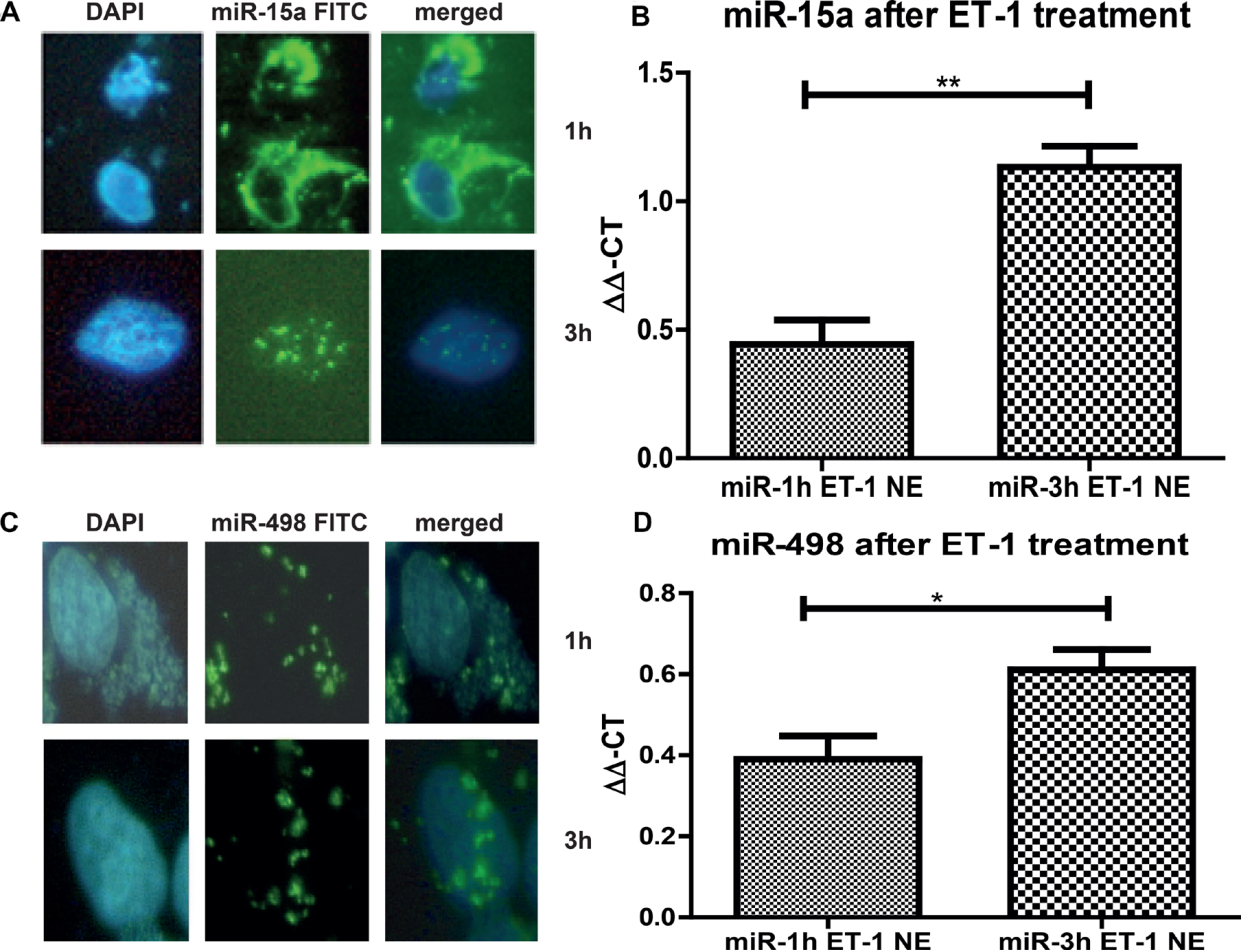
miR nuclear localizations 293T cells were grown on a Lab-Tek chamber slide, serum starved for 24 h and ET-1 treated for either 1 h or 3 h. For visualization of the nucleus DAPI stain was performed. (**A**) In parallel, cells were transfected with a FITC-labeled miR-15a. Cells were acetone/methanol fixed on the slide and analysed. After 1 h most of the miR is located in the cytoplasm, however after 3 h, over 60% of all counted cells show a translocation of the FITC miR-15a into the nucleus. (**B**) After ET-1 treatment of cells for 3 h, nuclear extracts were isolated and qRT-PCR for miR-15a was performed. Nuclear extracts from transfected control cells were used for normalisation and the ∆∆-ct method was used for evaluation. (**C**) Cells were grown and stimulated as described before. Cells were transfected with a FITC-labeled miR-498. After 3h treatment with ET-1, translocation of the miR-498 into the nucleus was found in more than 60% of cells. (**D**) Cells were either untreated or treated with ET-1 for 1 h or 3 h. Nuclear extracts were isolated followed by miR isolation with miRNeasy kit. qRT-PCR of the separated fractions was performed. As normalization, untreated cell extracts were used. After ET-1 stimulation for 3h a significant increase in miR-498 level in the nuclear fraction was detectable (*p* < 0.001).

**Figure 3:**
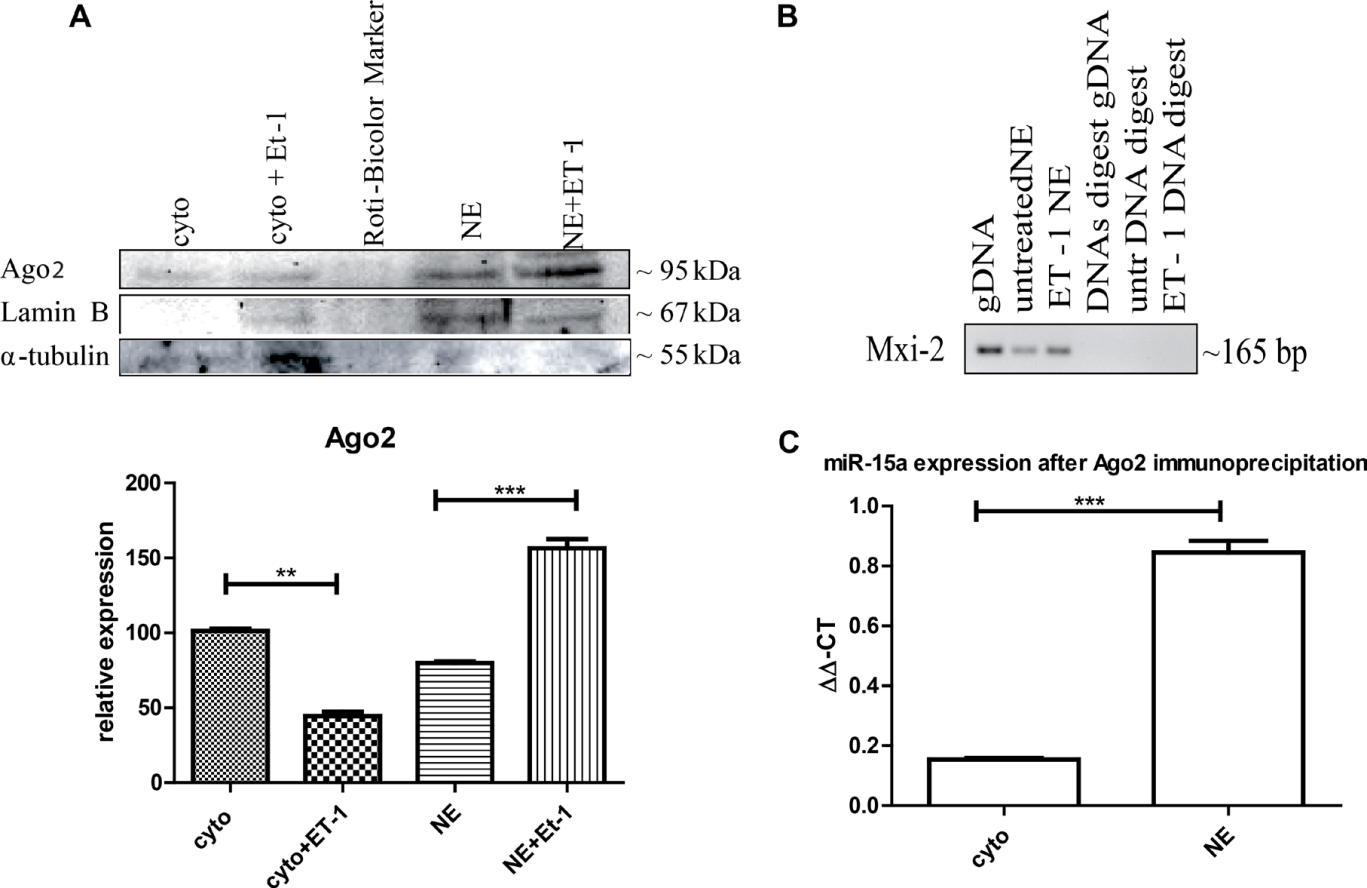
Ago2 involvement in miR:DNA interaction (**A**) Either cytoplasmic (cyto) or nuclear extracts (NE) were isolated from ET-1 treated (3 h) and untreated cells and Western blot analysis was performed using specific antibodies. Ago2 is present in the cytoplasm and the nuclear fraction but significantly increased in the nucleus after ET-1 treatment. (^***^p < 0.001, ^**^p < 0.01, ^**^p < 0.05) (**B**) Ago2-immunoprecipitation of the nuclear fraction, followed by DNA extraction and a PCR with Mix-2 specific primers. DNA specificity was demonstrated using a DNase digest. (**C**) For Ago2-immunoprecipitation, we treated 293T cells with ET-1 for 3 h and isolated the different cellular fractions, incubated them with an A/G agrosed beads coupled Ago2 antibody. From the different fractions the miR was isolated with a miRNeasy kit, followed by a qRT-PCR with specific miR-15a primers.

To this end, we needed to show translocation from the cytoplasm into the nucleus. To visualise the translocation of miRs in the nucleus, we transfected 293T cells with a FITC-labelled miR-15a and FITC-labelled miR-498 and treated them with Endothelin-1 (ET-1), since ET-1 is responsible for the translocalisation of miRs into the nucleus (Figure 2). After 1 hour the FITC signals were predominantly detectable in the cytoplasm (Figure 2A and 2C, upper lane, and Figure 2B and 2D, first column), but after 3 hour treatment the FITC miRs translocated into the nucleus (Figure 3A and 3C, lower lane, and Figure 2B and 2D, second column). The increase of FITC signal was statistically significant for both miRs. We have shown that both analyzed miRs accumulation into the nucleus after ET-1 induction.

### Nuclear location of Ago2

One known way for nuclear translocation of miRNAs is cytoplasmic binding Ago2 and translocation of Ago2 into the nucleus [20]. We were able to detect the presence of Ago2 in the cytoplasm as well as in the nuclear fraction, with an increased concentration in the nuclear fraction after ET-1 treatment (Figure 3A).

### Ago2 supports the interaction of miR:DNA

We furthermore performed Ago2 immunoprecipitation of the nuclear fraction, isolated the DNA and implemented a PCR with Mxi-2 specific primers (Figure 3C). To demonstrate that the shown signal is on DNA level, we conducted a DNase digest, again indicating that the interaction takes place on DNA level. Furthermore, we investigated whether Ago2 interacts with Mxi-2 DNA as well as with miR-15a in the nucleus. We conjugated Ago2 with agarose beads, isolated the DNA and performed an immuopreciptiation followed by a PCR with Mxi-2 specific primers. Shown in Figure 3B Ago2 does indeed co-immunoprecipitate with DNA. For miR-15a Ago2 interaction, we again used the conjugated Ago2 and isolated the miRs from the nuclear extract. Then, we again performed an immunoprecipitation followed by a real-time PCR for miR-15a. Figure 3C shows that there is an increase in the nuclear extract. Thus, Ago2 interacts with the newly identified miR:DNA complex.

### C-terminal truncated proteins and specific regions

**Figure 4:**
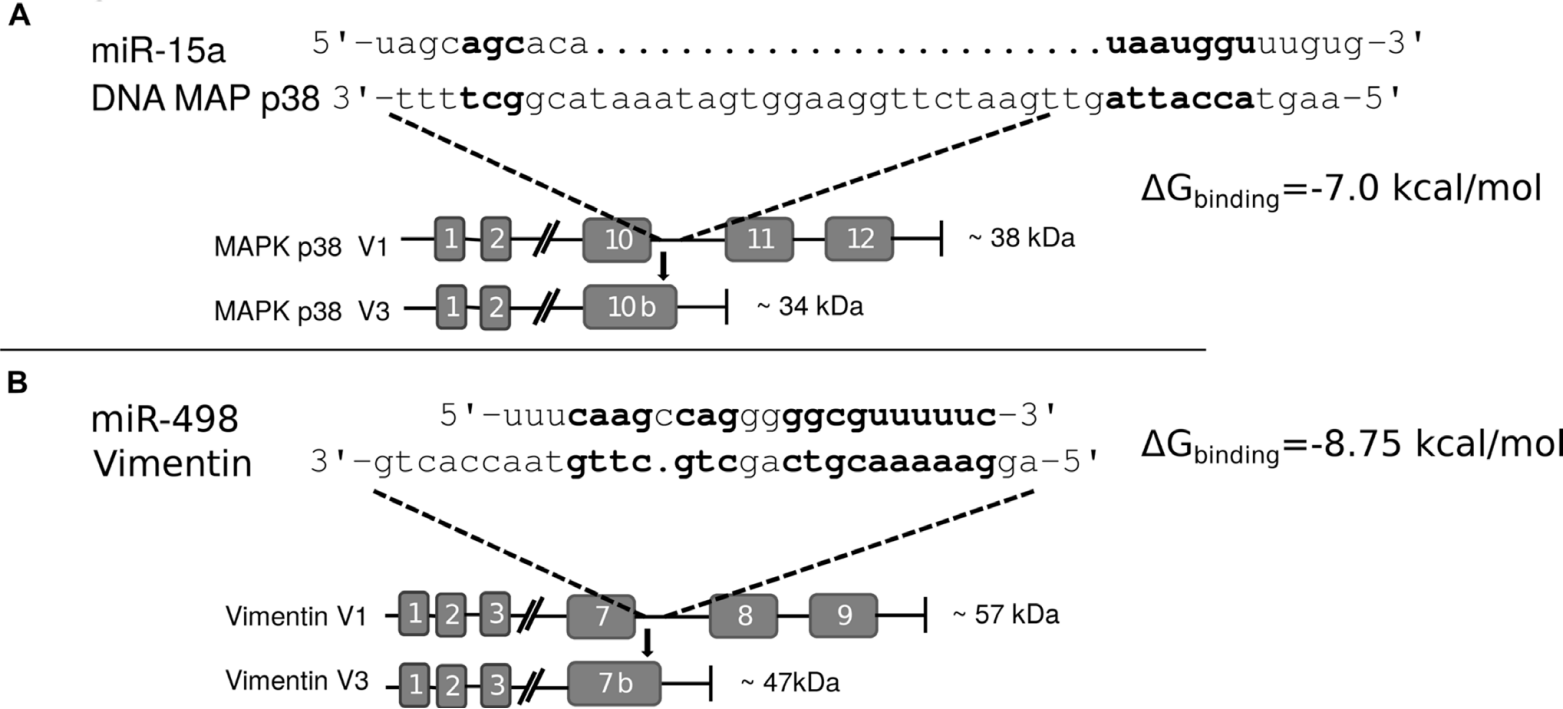
Interaction sites of miR and C-terminal truncated genes (**A**) Interaction site of miR-15a and the C-terminal end of Mxi-2 Bold letters indicate hypothetical base pairing interactions. (**B**) Interaction site between miR-498 and Vimentin variant 3 (Vim3) by comparing the miR sequence with the unique C-terminal ending of the truncated Vimentin version. Bold letters indicate the hypothetical interaction site. In both Figures the expected protein size (kDa) size is shown as well as the additional unique C-terminal ending of the proteins.

Comparing the two truncated proteins, Vim3 and Mxi-2, we “identical” an truncation behaviour. Both proteins were C-terminal truncated and have an identified extraordinary C-terminal ending. In both proteins, part of the last exon following intron is translated into amino acids. We compared the C-terminal endings with the overexpressed miRs found in the associated tumors and recognize binding homologous (Figure 4), but not in the classical manner by binding over the seed region only.

### Interaction of miR with DNA

Computationally looking for possible binding sites for the miRs on the respective pre-mRNAs, we did not find any putative binding sites. However, when looking at a possible miR:DNA interaction, we found that there were possible binding sites (Figure 4).

**Figure 5:**
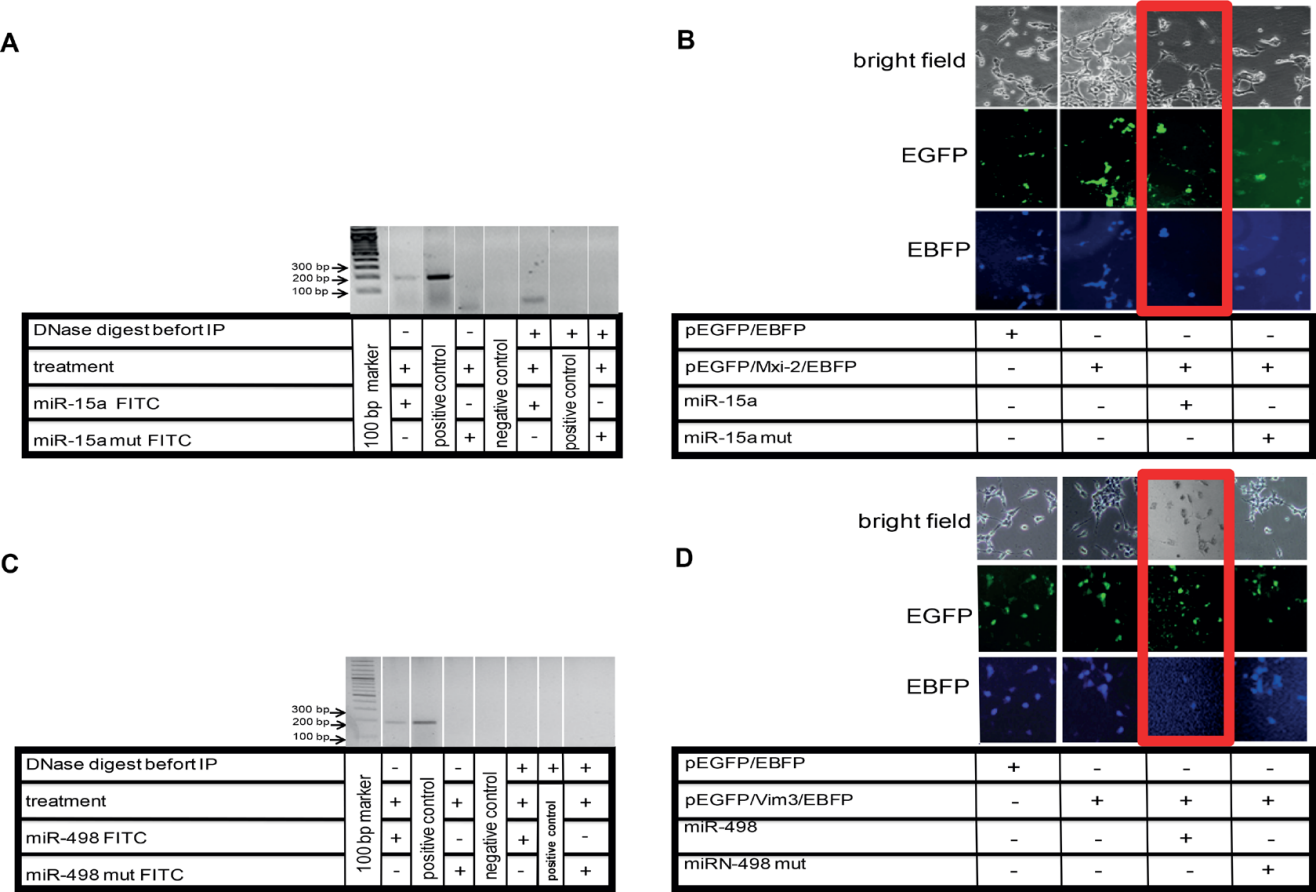
Visualization of miR:DNA interaction (**A**) Cells were transfected with FITC-miR-15a oligonucleotide and treated with ET-1 for 3 h. For determination of the interaction site, total FITC-oligonucleotide sequence was mutated (miR-15a mut FITC). DNA extraction was performed before and after treatment of cells with ET-1, followed by immunoprecipitation (IP), PCR was performed with Mxi-2 specific primers (Table1). To demonstrate that DNA binding of miR was involved in this interaction, DNase digestion (RNase-free) was performed before IP. As negative control, aqua dest. was used. DNA from cells without IP were used as positive control. (**B**) Double fluorescence vector Mxi-2 constructs for visualization of miR-15a and Mxi-2 interaction. A vector with an N-terminal pEGFP, a multiple cloning site and C-terminal EBFP was designed as described (pEGFP/EBFP). Its correct function was confirmed (first row). The PCR Mxi-2 product was cloned into pEGFP/EBFP. The final product was a pEGFP/Mxi-2/EBFP vector (second row). After transfections with miR-15a, the blue fluorescence disappears in nearly 80% of all cells, indicating that miR-15a binding leads to a loss of the C-terminal blue fluorescence signal (3rd row, red Box). Using mutated miR-15a only a 10% reduction was detectable (last row). (**C**) Cells were transfected with FITC-miR-498 oligonucleotide. To determine the interaction site, the FITC- oligonucleotide sequence was mutated (miR-498 mut FITC). DNA extraction was performed before and after treatment of cells with ET-1, followed by immunoprecipitation (IP), PCR was performed with Vim3 specific primers (Table 1). A DNase digestion was performed before IP. As negative control, water was used. (**D**) Double fluorescence vector Vim3 constructs for visualization of miR-498 and Vim3 interaction were employed. A vector was designed as already described (pEGFP/EBFP). The functionality was shown by the appearance of both signals (first row). The final product was a pEGFP/Vim3/EBFP vector, which shows the presence of the green and blue fluorescence (second row). After transfections with miR-498, the blue fluorescence disappears in nearly 70%, indicating that miR-498 binding leads to a loss of the C-terminal blue fluorescence signal (3rd row, red Box). Using a mutated miR-498 oligonucleotide (miR-498 mut) <15% signal reduction was found (fourth row).

To prove a possible interaction between miR and DNA, we transfected cells with FITC labeled miRs (15a or 498) and performed immunoprecipitation of these marked miRs. After this, DNA isolation and PCR with specific target gene primers showed that an interaction between the miRs and the DNA takes place (Figure 5A and 5C). Partial mutation of the miR sequences lead to signal loss and thus showed that these interactions are sequence specific. Moreover, the calculated binding energy for miR and DNA of either the Mxi-2/miR-15a (−7.0 kcal/mol) or the Vim3/miR-498 (−8.75 kcal/mol) complex revealed possible interaction (Figure 4).

**Table 1:** Primer and vector sequences

Gene	Sequence
miR-15a	Forw 5′-UAGCAGCACAUAAUGGUUUGUG-3′
FITC- miR-15a	UAGCAGCACAUAAUGGUUUGUG
5s rRNA	Forw 5′- GGCCAUACCACCCUGAACGC-3′
Mxi-2	Forw 5′-GACTCAGATGCCGAAGAT-3′ Rev 5′-TCAACTAATGGTACTTTATTTGG-3′
Mxi-2 (intron longer)	Forw 5′-GACTCAGATGCCGAAGAT-3′ Rev 5′-AGCCGTATTTATCACCTTC -3′
Mxi-2 (for vector)	Forw 5′-CTCGAGATGTCTCAGGAGAGGCCCAGC-3′ Rev 5′- CCATGGTCAACTAATGGTACTTTATTT-3′
Mxi-2 cc mut for vector	Forw 5′-CTCGAGATGTCTCAGGAGAGGCCCAGC-3′ Rev 5′- CCATGGTCAACTAAT**AA**TACTTTATTT-3′
Mxi-2 atggtaat mut for vector	Forw 5′-CTCGAGATGTCTCAGGAGAGGCCCAGC-3′ Rev 5′- CCATGGTCAAC**GCCGAAG**ACTTTATTT-3′
MAPK p38 (exon 8-11)	Forw 5′-GATGCATAATGGCCGAGCT -3′ Rev 5′-CAAGCATCTTCTCCAGCAAGTC -3′
gg mut. miR- 15a	UAGCAGCACAUAAU**AA**UUUGUG
FLANK mut. miR-15a	**CGUAGUAGAG**UAAUGGU**CCUCU**
mut. miR-15a	UAGCAGCACA**GCCGAAG**CUGUG
miR-498	UUUCAAGCCAGGGGGCGUUUUUC
mut miR-498	UUUCAA**AUUGAAAA**GCGUUUUUC
ß-actin	Forw 5′-TTGGCAATGAGCGGTTCCGCTG-3′ Rev 5′-TACACGTGTTTGCGGATGTCCAC-3′

Our results and the calculated energy values show that the interaction between miRs and DNA is feasible.

### miR interaction with DNA leads to intron retention

To show that miR:DNA interaction leads to splicing-terminal truncation, we designed a double fluorescence vector with a green fluorescence protein at the C-terminus and a blue fluorescence protein at the N-terminus. Between these fluorescence proteins the multiple cloning site is located, into which either the Mxi-2 or the Vim3 sequence was cloned. Every positively transfected cell shows a green and a blue fluorescence signal. The blue signal disappears in case of an interaction between the miRs and the vector (Figure 5B and 5D). The mutated miR sequences show the same signal intensity for green and blue, showing that the regulation is sequence specific.

### Genome-wide computational information

**Figure 6:**
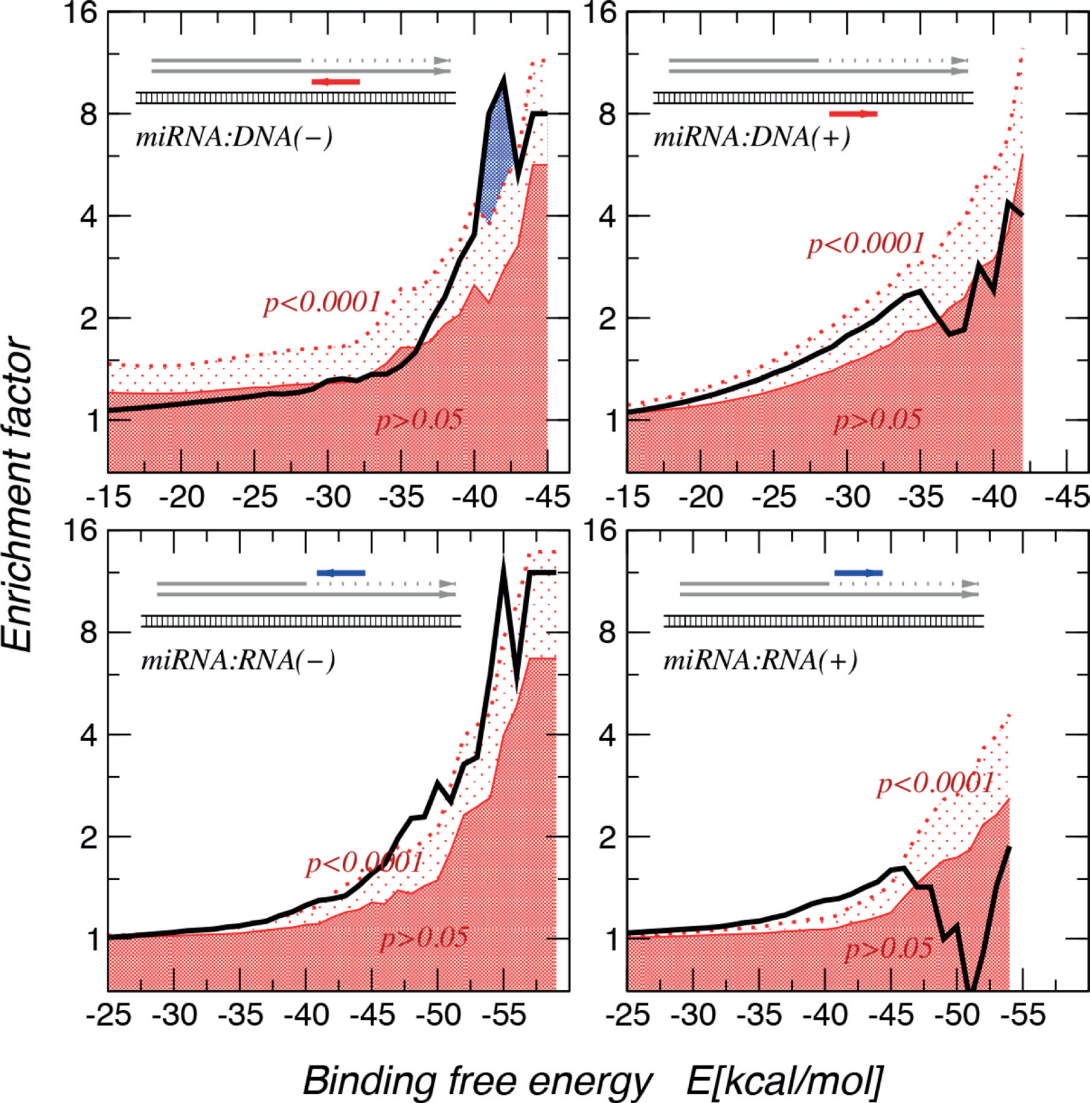
Enrichment of miR:target interactions with binding free energies The thick black line denotes the empirical data. The dark red area indicates enrichment values that are significant at less than *p* = 0.05 level, in the area above the dotted red line enrichment are significant at least at the *p* = 0.0001 level. The largest enrichment of very stable interactions are observed for miR:DNA interactions. The interactions seem to be directional, showing a strong preference for the directionality in which miR is complementary to the mRNA or DNA(−) strang.

Having calculated binding energies of these MAPK14 gene:miR-15a and Vimentin gene:mir-498, a genome wide computational analysis was performed to gain evidence whether this is a general mechanism beyond our two exemplary miRs. A statistically highly significant enrichment (*p* < 0.0001) of very stable interactions when using an approximate energy model [19] to match native miR sequences with the near-splice site miR:RNA and miR:DNA interactions was observed, showing much stronger and more significant enrichments for miR:DNA interactions (Figure 6). In order to identify miRs that show higher binding effects, we investigated the distribution of binding energies of miRbase target sites (regarding to the theory of C-terminal, intron included truncation) compared to their shuffled counterparts. A Kolmogorov-Smirnoff test for all distributions to check whether the energy distribution of the miRbase miR binding sites is significantly different and lower than the background distribution was performed. 66 miRs with a *p*-value < 0.01, a minimum number of target sites of 20, and at least one target site with a binding energy <−30 kcal/mol are reported here (Supplementary Figure 1). In Supplementary Figure 2 the top 5 miRs and target sequences were shown as well as the binding energy levels. This indicates a selective pressure, and thus we concluded that theses interaction possibly lead to a transcriptional stop.

### miRs and DNA interaction sites

**Figure 7:**
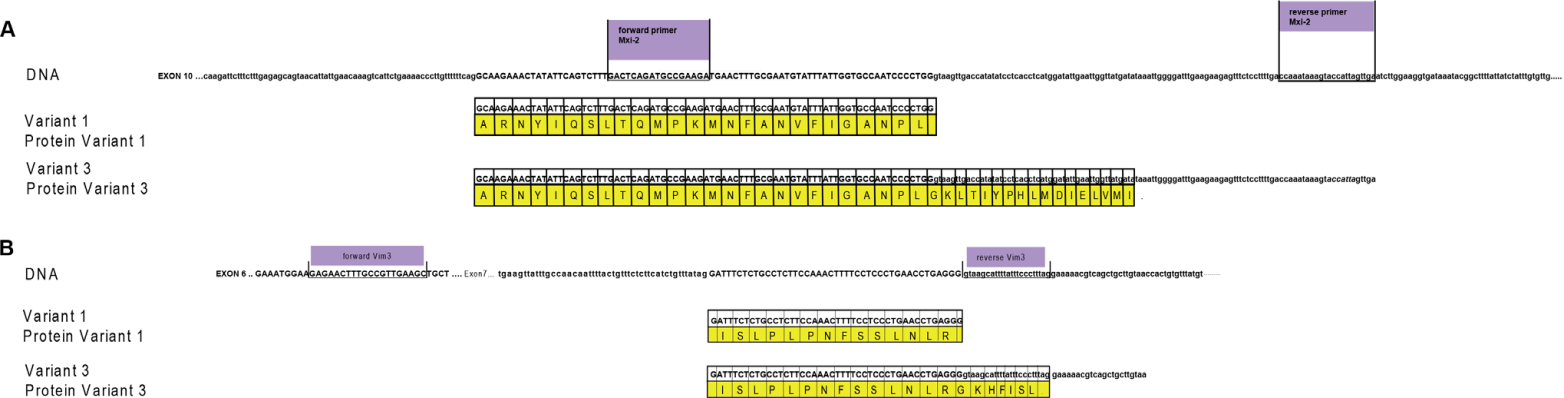
miRs and DNA interaction sites (**A**) Homology between variant 1 and variant 3 (Mxi-2) of the MAPK14 (MAPK p38α), starting at exon 10. Upper part shows the DNA sequence of MAPK p38α, the primer sequences are underlined and labelled with violet boxes. Small letters indicate intron regions and bold capital letters indicate exon regions. The yellow box presents the amino acid code; the point the stop codon. (**B**) Homology between variant 1 and variant 3 (Vim3) of Vimentin, starting at exon 6. The upper part shows the DNA sequence of vimentin, the primer sequences are underlined and labelled with violet boxes. Small letters indicate intron regions and bold capital letters indicate exon regions (green box). Yellow box: amino acid code.

To visualize the binding sites of miR-15a and mir-498 Figure 7 was depicted. Homology between variant 1 and variant 3 (Mxi-2/Vim3) of the MAPK14 and Vimentin is shown, the unique C-terminal ending includes the intron region, shown here in small capital letters.

## DISCUSSION

### miR-498/Vim3 and miR-15a/Mxi-2 are examples of a novel kind of regulation by miRNAs

We have shown that there is a regulation of isoform usage that is facilitated by miRNAs. In the cases of the biomarkes Vim3 and miR-498 for oncocytoma as well as miR-15a and Mxi-2 for RCC, we were able to show that the coincidence of the upregulation is not a mere co-occurrence. In both cases, the miR is necessary for the truncated isoform to exist. Furthermore, we showed that the miR is actually binding to DNA. Computational experiments pointed to binding to the antistrand DNA, as binding is energetically more favourable there.

The interaction between the miR and DNA leads to transcriptional stop, which is to in opinion necessary, since otherwise, the splice donor site on the following exon is still present and the spliceosome will perform the usual splicing mechanism.

**Figure 8:**
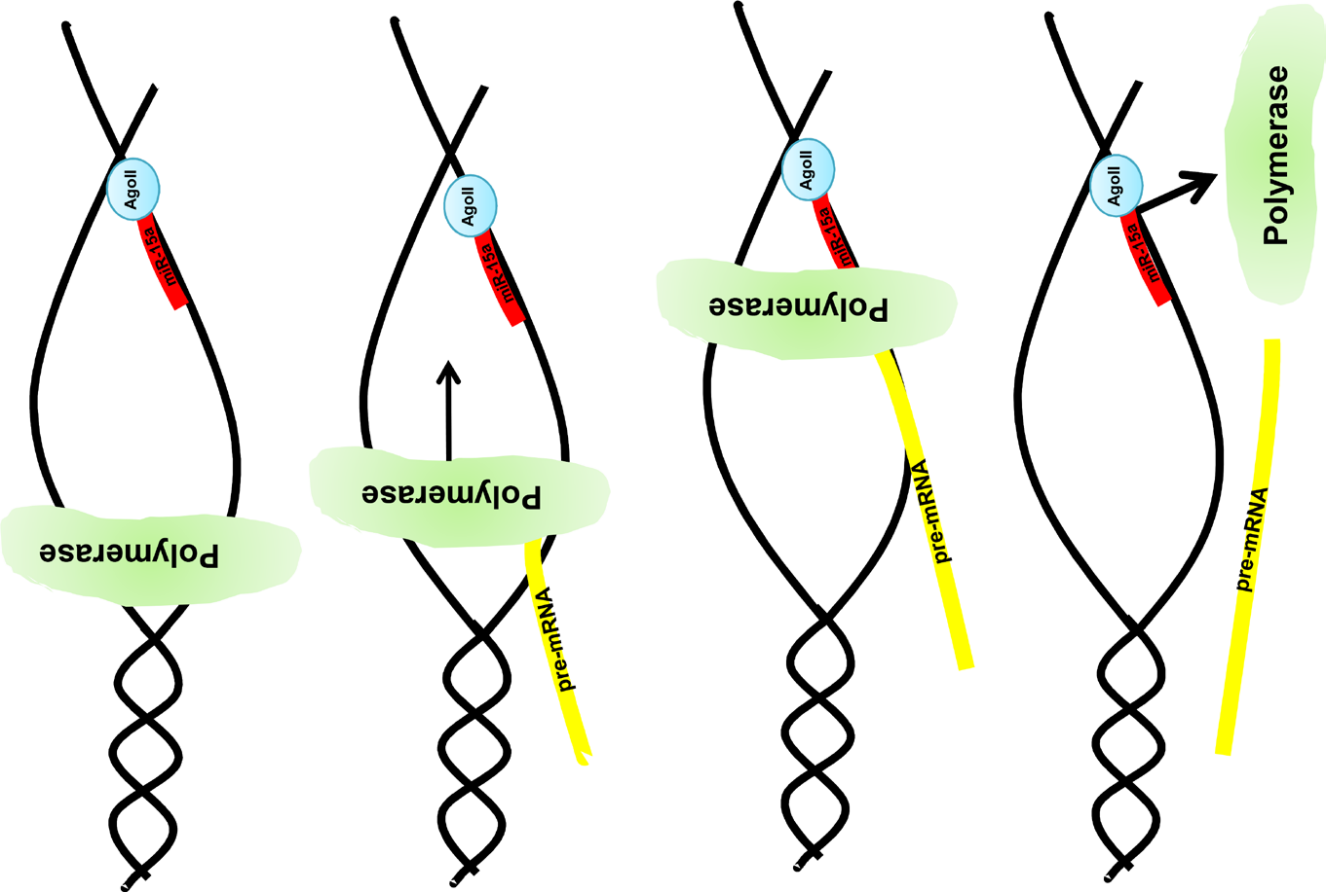
Graphical illustration of the miR:DNA interaction After opening the DNA strand nuclear located e.g. miR-15a in combination with the Ago2 binds to the uncoded strand of the given gene e.g. MAPK14. Due to this complex formation the DNA polymerase stops the pre-mRNA synthesis and a truncated pre-mRNA is produced which results in a truncated protein (e.g. Mxi-2).

The novel interaction mechanism depicted in Figure 8 has at least two implications.

The first feature may be the difference in regulation of pre-mRNAs being truncated versus their regulation at the mRNA level. Regulation of the mRNAs in the RISC (RNA-induced-silencing-complex) is believed to be essentially based upon the presence of a 3′ polyA tail deadenylation [21], either followed by 3′- to 5-′ exonucleolytic degradation by the exosome or alternatively being decapped by decapping enzymes and degraded by exoribonuclease. The second feature, since this 3′ polyA tail is missing, we hypothesize that truncated pre-mRNAs and the resulting mRNAs could successfully escaped this regulation through the RISC and is directly translated. Although it is known that mRNAs without poly A tail are not very stable and that the translation into protein is not frequent it is known from histones, that mRNAs can exist without a polyA tail [22].

In conclusion, miRs can bind to the non-coding DNA strand and induce a transcriptional stop that results in the production of proteins with truncated C-termini. Computational experiments suggest that this mode of miR function might be used much more often than in the two cases shown here. If this is the case, this can have a profound influence on the human protein repertoire, with many putative truncated isoforms that may have influence in many diseases.

## MATERIALS AND METHODS

### Cell culture

293T cells (www.DSMZ.de, P/N ACC 635) were ccultured in Dulbecco’s Modified Eagle Medium (GIBCO, Darmstadt, Germany), supplemented with 10% FCS (PAN Biotech GmbH, Aidenbach, Germany) and 1% Penicillin/Streptomycin (GIBCO, Darmstadt, Germany) at 37° C and 5% CO_2_. For all experiments, cells were pre-incubated for 24 h with serum-free DMEM.

### Cell treatments

Cells were treated with endothelin-1 (ET-1) (50 nM) for different time points. The cells were serum starved for 24 h before treatments.

### miR:DNA-immunoprecipitation

Before performing the immunoprecipitation, cells were transfected with FITC-labeled miR oligos, either with or without mutation and then stimulated with ET-1. For FITC-miR-immunoprecipitation, an anti-FITC mouse antibody (Santa Cruz, Heidelberg, Germany) was incubated with an A/G PLUS-agarose antibody (Santa Cruz) on ice for 2 h. Afterwards the whole DNA, isolated either from treated cells or appropriate controls, was incubated on ice under the same conditions as described below. The samples were washed twice with 1xPBS and a PCR was performed. As negative control only agarose beads, an unlabeled miR and FITC-labeled mutated miR were used. Half of the PCR product was loaded on a 2% agarose gel and the other half was used for Sanger sequencing.

### Ago2 interaction

Ago2 antibody (Santa Cruz, polyclonal) was used. Cell fractions were separated according to the nuclear extraction protocol. The different fractions were incubated for 1 h on ice with the Ago2 antibody, followed by an incubation period on ice for 2 h with A/G PLUS-agarose antibody (Santa Cruz). Afterwards either the DNA (Mxi-2 detection/PCR) or the miR was isolated and PCR was performed.

### Sanger sequencing

The PCR product from the FITC-miR- immunoprecipitation was prepared for Sanger sequencing according to the BigDye^®^ V3.1 Sequencing Kit (Applied Biosystems Inc, Forster City, USA) protocol. The product was sequenced by the Cologne Center for Genomics and analysed with the 4Peaks V1.7.2 (The Netherlands Cancer Institute, Amsterdam, The Netherlands) and BLAST [23].

### DNA extraction

The QIAamp DNA mini kit (Qiagen, Hilden, Germany) was used for DNA extraction of transfected 293T cells, according to the manufacturer’s protocol.

### DNase digest

The digest was performed before FITC-miR-immunoprecipitation, using the New England Biolabs DNaseI (RNase-free) enzyme (New England Biolabs, Frankfurt, Germany) according to the provided protocol.

### miR isolation

The RNeasy FFPE kit (Qiagen, Hilden, Germany) was used for miR isolation of the FFPE samples, according to the manufacturer’s protocol. RNA quantification was accomplished using the NanoDrop technology (Thermo Scientific, Oberhausen, Germany). The miR was isolated from cells with the miRNeasy Kit (Qiagen, Hilden, Germany), according to the manufacturer’s protocol.

### Fluorescence imaging

A Nikon F601 camera (Nikon GmbH, Duesseldorf, Germany) connected to an Olympus microscope DP71 (Olympus, Hamburg, Germany) was used for fluorescence imaging. The CellP^2.5 Program, Olympus soft imaging solutions (Olympus) was used to evaluate the fluorescence pictures.

### Transfection

Subconfluent cells were grown in a monolayer on 6 well plates. 100 nM oligos were always used for transfection. The oligo was diluted in 100 µl OptiPAN (PAN Biotech, Aidenbach, Germany) containing 6 µl FuGENE HD (Roche, Mannheim, Germany). The transfection was performed according to the FuGENE HD protocol. The whole suspension was drop-wise re-suspended in 900 µl OptiPAN medium including 50 nM ET-1. After 1 and 3 h the samples were analyzed and DNA was isolated as described in DNA extraction. A double-labelled vector was constructed for pEGFP/EBFP analysis. The commercially available vectors pEGFP and pEBFP (Clontech, Saint-Germain-en-Laye, France) were digested with *AseI* and *BamHI-HF* (New England Biolabs). The digested product of the pEGFP sized ∼1350 bp was cloned in the resulting ∼4200 bp pEBFP. The final product was a green/blue vector with a multiple cloning site (MCS) between both signals. The green/blue vector was digested with *XhoI and KpnI-HF* (New England Biolabs) and the PCR amplified, *XhoI and KpnI-HF* digested Mxi-2 product (NM_139013, position 482 to 1431) was cloned (pEGFP/Mxi-2/EBFP). Furthermore, two mutated versions of this vector were also generated (pEGFP/Mxi-2 gc mut/EBFP and pEGFP/Mxi-2 atggtaat mut/EBFP). Analog to pEGFP/Mxi-2/EBFP the vector pEGFP/VIM3/EBFP was constructed. Cells were either transfected with: miR-15a oligo alone (100 µM), the pEGFP/EBFP vector (1 µg), pEGFP/Mxi-2/EBFP (1 µg) or pEGFP/Mxi-2/EBFP plus miR-15a oligo (1 µg + 100 µM). Cells were analysed with the Cell^P program (Olympus) 24 h and 48 h after transfection. All experiments were performed with four different clones.

### Nuclear extract and cytoplasmic isolation

Nuclear and cytoplasmic extracts were isolated from treated cells or controls according to the manufacturer’s protocol (nuclear extraction kit, Active motif, La Hulpe, Belgium). Protein content was assayed with the Bradford protein assay (Bio-Rad, Munich, Germany), with bovine serum albumin as standard.

### Renal carcinoma and oncocygtoma samples

Formalin-fixed and paraffin embedded (FFPE) human samples from the archives of the Department of Pathology, University Hospital of Cologne, Germany were used. Histologic evaluation was based upon analyses by a staff pathologist (J.W.U. Fries) using Hematoxilin-Eosin stained paraffin-sections.

Since human materials (RCC, Onco) were used (12 and 6 per group), procedures were followed as outlined in accordance with ethical standards formulated in the Helsinki Declaration 1975, with pre-approval by the local Ethics Committee at the University Hospital (reference number: 09-232).

### RT-PCR

The cDNA was obtained from 250 ng RNA using random primers and SuperScript III reverse transcriptase according to the manufacturer’s protocol (Invitrogen, Darmstadt, Germany). The RT-PCR was performed as previously described [24, 25].

The RNeasy FFPE kit from Qiagen was used for miRNA isolation of the FFPE samples, according to the manufacturer’s protocol. RNA quantification was accomplished using the NanoDrop technology. The miRNA was isolated from cells with the miRNeasy Kit from Qiagen, according to the manufacturer’s protocol.

### Quantitative real-time PCR (qRT-PCR)

1 µl of the cDNA (transcribed from 250 ng RNA) was used for real-time PCR analysis. The experimental settings were as previously described [24, 25]. For quantitative analysis, β-actin was measured. All samples were normalized to ß-actin as reference gene. All experiments were done in triplicate. When working with miRs, instead of the ß-actin, 5s rRNA was used for normalization. The ΔΔC_T_ method was used for calculation as outlined in User Bulletin 2 (PE Applied Biosystems, Forster City, USA). Untreated cells were used as controls. For the statistical significance of qRT-PCR values student’s *t*-test was applied.

### miR presence in the nucleus

These values were calculated as followed: the difference between 1 and 3h ET-1 treated and untreated cells were calculated first, the cytoplasmic levels were used as baseline levels and calculated against the values resulting from the NE fraction. Afterwards the miR-15a or the miR-498 was compared between 1 and 3 h. In both cases a statistical significant increase was detectable.

### Statistics

All experiments were performed in a set of three independent experiments. The GraphPrism 5 (Graphpad software, La Jolla, USA) program was employed for the statistical analysis. For statistics a Student’s paired *t-*test was utilized. The ImageJ [26] lab version was used to measure densitometry. A student’s *t*-test was applied for statistical analysis with the result that all differences between cytoplasmic (cyto) vs nuclear extracts (NE) have a statistical significance (^***^p < 0.0001, ^**^p < 0.001,^*^p < 0.05).

### Western blot analysis

Western blot analysis was performed as previously described [24, 25]. For the analysis of Mxi-2, a commercially available antibody from nanoTools (clon 2F2) was used and tested for specificity with the provided positive control lysate (A431). For identification of the nuclear extract fraction an antibody against alpha-tubulin, (Santa Cruz, clon TU-02), and for the cytoplasm an antibody against Lamin B (Cell signaling, clon D4Q4Z), was used. Ago2 antibody (Santa Cruz, H-300) was applied according to the manufacturer’s protocol.

### Bioinformatics

We used GENCODE19 annotation [27] of the human genome to extract all loci that are annotated as both intron and exon at the same position. After removing loci that also intersect other annotation items and first exons, we obtained 51,987 locations. The subsequence extending from 10 nt outside the intersection to 40 nt into the intersection is used as a potential target for the small RNA. For 21,393 loci the overlap is shorter than 100 nt. In this case we used the entire short intron plus 10 nt on either side as the potential target. All human miR sequences listed in miRBase release 20 [28] were used as possible interaction partners. To keep computational resources manageable we first ran RNAplex [29] with the option -f 1 to estimate binding energies and location of interactions using a simplified energy model. Candidate interactions with an RNAplex energy E <−15 kcal/mol were kept and re-evaluated with RNAduplex [30] for more accurate RNA:RNA binding energies. For RNA:DNA pairing, we used a variant of this tool that computes miR:DNA hetero duplexes [31] and a more stringent RNAplex filter of at least E <−30 kcal/mol. As expected from the large number of candidate pairings, we obtained a very large number of candidate interactions. To assess their significance we computed the enrichment of observed highly stable interactions compared to the background estimated by recomputing the interactions with permuted pseudo-miRs. The latter were obtained by dinucleotide shuffling using Peter Clote’s implementation Dishuffle of the Altschul-Erikson dinucleotide shuffle algorithm [23]. In order to determine whether the interactions are strand specific or not, we distinguished between the interaction of miRs in the complementary and the parallel direction with regard to the GENCODE19 mRNAs.

## SUPPLEMENTARY MATERIALS FIGURES


