# Supplementary material for: Beyond the 3′UTR binding–microRNA-induced protein truncation via DNA binding
Source: Oncotarget. 2018 Aug 28;9(67):32855–67. doi: 10.18632/oncotarget.26023 (PMC6132356; doi:10.18632/oncotarget.26023)
Supplement: Supplementary file 1 [file oncotarget-09-32855-s001.pdf]

Beyond the 3'UTR binding–microRNA-induced protein truncation via DNA binding

SUPPLEMENTARY MATERIALS

Micro RNA	bind. sites < − 30	total binding sites	Kolmogorov-Smirnov p-value	Mean Δ E	Δmean(shuff.-miRBase)
hsa-miR-1207-5p	8	19,868	5.35E-03	-18.27	0.05
hsa-miR-1226-5p	7	17,798	3.19E-04	-17.65	0.07
hsa-miR-1228-5p	6	11,602	2.33E-60	-17.71	0.45
hsa-miR-1233-5p	2	14,049	2.68E-03	-17.80	0.07
hsa-miR-1237-5p	37	12,834	1.94E-07	-18.59	0.15
hsa-miR-1273h-5p	1	3,319	1.59E-09	-16.78	0.21
hsa-miR-1469	37	13,728	1.63E-09	-18.44	0.21
hsa-miR-1909-3p	1	10,844	5.75E-03	-17.29	0.02
hsa-miR-1972	3	5,291	3.81E-11	-16.88	0.23
hsa-miR-3196	1	11,911	6.70E-25	-18.10	0.29
hsa-miR-3197	1	9,874	4.11E-03	-17.30	0.03
hsa-miR-328-5p	97	22,147	6.39E-05	-18.87	0.04
hsa-miR-3620-5p	48	19,727	2.97E-16	-18.41	0.23
hsa-miR-3940-5p	1	7,528	2.67E-33	-17.43	0.31
hsa-miR-4298	1	11,465	2.54E-11	-17.58	0.19
hsa-miR-4459	5	14,166	1.50E-35	-17.65	0.32
hsa-miR-4488	4	9,467	3.15E-08	-18.02	0.12
hsa-miR-4508	1	7,273	3.59E-05	-17.86	0.12
hsa-miR-4632-5p	1	15,535	2.27E-05	-17.66	0.07
hsa-miR-4649-5p	2	13,392	3.70E-14	-17.67	0.16
hsa-miR-4656	2	18,300	1.79E-25	-17.85	0.19
hsa-miR-4665-3p	25	10,241	4.97E-04	-18.13	0.11
hsa-miR-4674	1	8,352	2.23E-03	-17.63	0.08
hsa-miR-4685-5p	2	19,846	8.00E-17	-17.77	0.15
hsa-miR-4728-5p	1	10,661	9.92E-03	-17.81	0.04
hsa-miR-4739	63	23,215	2.25E-21	-18.79	0.22
hsa-miR-4749-5p	2	11,811	1.12E-24	-17.78	0.26
hsa-miR-4758-5p	2	13,522	2.34E-43	-17.55	0.29
hsa-miR-4787-5p	28	12,201	6.79E-10	-18.23	0.23
hsa-miR-5006-5p	1	9,393	4.68E-05	-17.08	0.09
hsa-miR-5585-3p	1	976	1.04E-171	-19.01	2.78
hsa-miR-602	1	7,578	2.53E-12	-17.11	0.16
hsa-miR-6089	576	27,100	1.99E-03	-19.49	0.11
hsa-miR-612	2	7,658	8.42E-04	-16.91	0.06
hsa-miR-619-5p	36	2,242	4.30E-147	-18.64	2.28
hsa-miR-6511b-5p	1	7,537	8.91E-04	-16.95	0.06
hsa-miR-6724-5p	71	21,705	3.50E-03	-18.73	0.06
hsa-miR-6726-5p	1	9,717	7.62E-03	-17.23	0.06
hsa-miR-6727-5p	14	20,744	3.53E-22	-18.27	0.22
hsa-miR-6732-5p	1	13,053	1.87E-08	-17.72	0.11
hsa-miR-6741-5p	1	9,994	1.87E-17	-17.12	0.20
hsa-miR-6743-5p	15	19,225	1.04E-13	-18.23	0.19
hsa-miR-6749-5p	16	16,667	2.30E-07	-18.23	0.16
hsa-miR-6750-5p	8	8,283	2.97E-03	-17.00	0.10
hsa-miR-6756-5p	81	25,680	2.01E-22	-18.84	0.24
hsa-miR-6765-5p	11	20,654	1.99E-24	-18.06	0.25
hsa-miR-6775-5p	138	24,037	3.45E-15	-18.94	0.19
hsa-miR-6782-5p	5	11,765	2.65E-18	-18.08	0.28
hsa-miR-6786-5p	34	17,146	9.93E-03	-18.42	0.06
hsa-miR-6789-5p	48	15,033	4.27E-10	-18.44	0.17
hsa-miR-6803-5p	138	20,286	5.67E-04	-18.88	0.10
hsa-miR-6812-5p	3	13,882	1.81E-08	-17.74	0.13
hsa-miR-6836-5p	2	14,879	1.34E-06	-17.68	0.11
hsa-miR-6848-5p	7	16,784	8.58E-04	-18.16	0.07
hsa-miR-6850-5p	2	10,179	1.26E-10	-17.60	0.14
hsa-miR-6858-5p	4	15,017	5.26E-26	-17.82	0.26
hsa-miR-6860	1	10,259	1.02E-26	-17.14	0.22
hsa-miR-6869-5p	1	4,191	2.63E-14	-17.32	0.27
hsa-miR-6879-5p	1	13,660	6.35E-15	-17.80	0.18
hsa-miR-6889-5p	1	6,566	8.64E-08	-17.22	0.19
hsa-miR-6894-5p	2	11,750	3.66E-03	-17.36	0.04
hsa-miR-7107-5p	3	15,531	1.36E-06	-17.90	0.11
hsa-miR-7111-5p	2	9,120	1.65E-04	-17.75	0.09
hsa-miR-762	143	22,549	3.19E-06	-18.91	0.13
hsa-miR-8089	7	15,800	4.92E-22	-18.10	0.28
hsa-miR-939-5p	3	17,736	3.62E-07	-17.91	0.11

Supplementary Figure 1: miRs listed with the highest number of DNA binding sites.

miRNA	Gene	Binding engery kcal/mol
hsa-miR-6089	SIX5	-38,85
hsa-miR-6089	CYP2W1	-39,15
hsa-miR-6089	RNF38	-39,5
hsa-miR-6089	PRODH	-42,75
hsa-miR-6089	LYNX1	-41,65
hsa-miR-6089	MSRB1	-39
hsa-miR-6089	CENPM	-38,2
hsa-miR-6089	WDR86	-40,6
hsa-miR-6089	MAML1	-38,7
hsa-miR-6089	STAU2	-39,7
hsa-miR-6089	SIRT3	-41,45
hsa-miR-6089	UBTF	-38,8
hsa-miR-6089	CDKN2D	-43,45
hsa-miR-6089	ZNF652	-38,65
hsa-miR-6089	CST3	-41,75
hsa-miR-6089	CDNF	-39,5
hsa-miR-6743-5p	MSLNL	-38,35
hsa-miR-6789-5p	DONSON	-38
hsa-miR-6789-5p	PLEKHJ1	-47,55
hsa-miR-6803-5p	CBLN1	-42,2
hsa-miR-6869-5p	FKBP1A	-38,65

Supplementary Figure 2: miRs with the highest number of DNA binding sites was chosen and exemplary some genes and the energy potential values were determined.
